# Correction: Adherence towards COVID-19 mitigation measures and its associated factors among Gondar City residents: A community-based cross-sectional study in Northwest Ethiopia

**DOI:** 10.1371/journal.pone.0256954

**Published:** 2021-08-26

**Authors:** Zelalem Nigussie Azene, Mehari Woldemariam Merid, Atalay Goshu Muluneh, Demiss Mulatu Geberu, Getahun Molla Kassa, Melaku Kindie Yenit, Sewbesew Yitayih Tilahun, Kassahun Alemu Gelaye, Habtamu Sewunet Mekonnen, Abere Woretaw Azagew, Chalachew Adugna Wubneh, Getaneh Mulualem Belay, Nega Tezera Asmamaw, Chilot Desta Agegnehu, Telake Azale, Animut Tagele Tamiru, Bayew Kelkay Rade, Eden Bishaw Taye, Asefa Adimasu Taddese, Zewudu Andualem, Henok Dagne, Kiros Terefe Gashaye, Gebisa Guyasa Kabito, Tesfaye Hambisa Mekonnen, Sintayehu Daba, Jember Azanaw, Tsegaye Adane, Mekuriaw Alemayehu

The last author’s name is spelled incorrectly. The correct name is: Mekuriaw Alemayehu.

## References

[1] Azene ZN, Merid MW, Muluneh AG, Geberu DM, Kassa GM, et al. (2020) Adherence towards COVID-19 mitigation measures and its associated factors among Gondar City residents: A community-based cross-sectional study in Northwest Ethiopia. PLOS ONE 15(12): e0244265. doi: 10.1371/journal.pone.0244265 33378332 PMC7773181

